# Severe systemic inflammatory response syndrome in patients following Total aortic arch replacement with deep hypothermic circulatory arrest

**DOI:** 10.1186/s13019-019-1027-3

**Published:** 2019-12-16

**Authors:** Jun Li, Lijing Yang, Guyan Wang, Yuefu Wang, Chunrong Wang, Sheng Shi

**Affiliations:** 1 Department of Anesthesiology, Fuwai Hospital, Chinese Academy of Medical Sciences, Peking Union Medical College, National Center for Cardiovascular Diseases, State Key Laboratory of Cardiovascular Disease, Belishi road 167, Xicheng District, Beijing, 100037 China; 2 Department of Anesthesiology, Beijing Tongren Hospital, Capital Medical University, Beijing, 100730 China

**Keywords:** Severe systemic inflammatory response syndrome, Total aortic arch replacement, Advancing age, Clinical outcomes

## Abstract

**Background:**

This cohort study aims to retrospectively investigate the incidence of severe systemic inflammatory response syndrome (sSIRS) in patients following total aortic arch replacement (TAR) under deep hypothermic circulatory arrest (DHCA) with selective cerebral perfusion and its effect on clinical outcomes.

**Methods:**

All patients who underwent TAR with DHCA were consecutively enrolled from January 2013 until December 2015 at our institute. sSIRS was diagnosed between 12 and 48 h postoperatively if patients met all four criteria of the SIRS definition.

**Results:**

Of the 522 patients undergoing TAR with DHCA, 31.4% developed sSIRS. Patients aged under 60 yr were characterized by a higher prevalence of sSIRS (OR = 2.93; 95% CI 2.01–4.28; *P* <0.001). Higher baseline serum creatinine (OR = 1.61; 95% CI 1.18–2.20; *P* = 0.003), concomitant coronary disease (OR = 2.00; 95% CI 1.15–3.48; *P* = 0.015) and extended cardiopulmonary time (OR = 1.63; 95% CI 1.23–2.18; *P* = 0.001) independently contributed to a greater likelihood of postoperative sSIRS onset, while the preferred administration of ulinastatin (OR = 0.69; 95% CI 0.51–0.93; *P* = 0.015) and dexmedetomidine (OR = 0.36; 95% CI 0.23–0.56; *P* < 0.001) attenuated it. Patients with sSIRS had a greater risk of developing postoperative major adverse complications compared with the no sSIRS group [56.7%(93/164) vs 26.8% (96/358), *P* < 0.001]. sSIRS was found to be a significant risk factor for major adverse complications (OR, 4.52; 95% CI, 3.40–6.01; *P* < 0.001). A significant difference was revealed in in-hospital death following TAR between the sSIRS group and the no-sSIRS group [4.88% (8/164) vs 1.12% (4/358), *P* = 0.019]. The Kaplan-Meier curve indicated that the time to discharge from the intensive care unit was significantly prolonged in the sSIRS group compared with patients without it (log-rank *p* < 0.001).

**Conclusions:**

sSIRS occurs commonly in patients following TAR with DHCA. There is an inverse association between age and sSIRS onset, whereby age over 60 yr can lower the risk of it. sSIRS development can increase the likelihood of major postoperative major adverse events.

## Introduction

Postoperative systemic inflammatory response syndrome (SIRS), discussed in some specific cardiovascular interventions such as transcatheter or open aortic valve repair [1–3], has received limited attention yet. Nevertheless, studies on the prevalence of inflammatory responses following open total aortic replacement (TAR) using 4-branch graft are lacking. Inflammation following TAR can be incurred by operative trauma, blood exposed to cardiopulmonary bypass (CPB), infection and hypothermia, although the combination of TAR and DHCA to treat aortic pathology with selective cerebral perfusion is technically preferable.

SIRS, a whole-body inflammation, has remained a primary clinical problem to be addressed despite significant improvement of strategies in diagnostics and therapy. As SIRS progresses, the incidence of morbidity, such as multi-organ failure and even mortality, will increase in cardiac [4, 5] and noncardiac settings [6, 7]. The inflammatory response has detrimental effects on cardiac function, and it arouses respiratory problems, hemostasis dysfunction, and kidney or liver injury. However, SIRS as a consequence of multiple coexisting factors has been controversial due to its high prevalence but low specificity for infection [8]. It is currently recognized that severe SIRS (sSIRS) is a more practical term for inflammation and has high accuracy to predict prognosis [2, 9].

We hypothesize that sSIRS following TAR with DHCA appears often because of the complex and risky surgical procedure and contact with CPB. This retrospective study carried out at our institute is intended to explore the incidence, independent predictors, and clinical outcomes of sSIRS in patients following TAR with DHCA.

## Methods

This cohort study was approved by the Ethics Committees of Fuwai Hospital. Informed consent from the participants was waived for the retrospective nature. All details of design, conception and conduct are listed in the following parts.

### Study population

There were a total of 546 charts of patients enrolled consecutively, who underwent TAR with DHCA from January 2013 to December 2015. The perioperative information was obtained from digital medical records at our center. Participants meeting one of the following were excluded: a) mechanical ventilation support before surgery; b) infection prior to surgery; c) death during surgery or the first 48 h postoperatively; d) precocious postoperative infection within the first 5 days; and e) record with incomplete data. The purpose of these exclusion criteria was to eliminate known causes of inflammation with a deflagration process, especially secondary to prolonged mechanical ventilation treatment and other infections. Finally, 24 patients were excluded, and valuable data on the population of 522 subjects were collected. Of these, 496 were all operated on for acute type A dissection, 26 for aneurysms and 60 for Marfan syndrome.

### Clinical definitions

SIRS was diagnosed on the basis of the existing American College of Chest Physicians/Society of Critical Care Medicine Consensus Conference if patients met at least two of the following: (1) white blood cell count <4 or > 12 (10^9^ /L); (2) heart rate > 90 bpm or partial pressure of arterial carbon dioxide (PaCO_2_) < 32 mmHg; (3) temperature > 38 or < 36 °C; (4) respiratory rate > 20 per minute [10]. The application of sSIRS was used if all four criteria were met. In our study, sSIRS was observed within 12–48 postoperative hours after intensive care unit (ICU) admission from the operating room. No evaluation of the occurrence of any criteria related to the first 12 h following repair was carried out, which aimed to avoid spurious findings produced by numerous inotropes, diuretic drugs and various fluid inputs achieved immediately in the postoperative setting. Finally, patients in our cohort were divided into the sSIRS group and the no-sSIRS group.

Emergent treatment at our institute was defined as surgery within 24 h of admission to the hospital. All patients routinely received both patient-controlled analgesia and continuous body rewarming during their treatment in the ICU. Prolonged ICU length was defined as a stay of greater than 7 days. The assessment of renal failure before and after TAR was based on stage III in the Kidney Disease: Improving Global Outcomes (KDIGO) criteria. Hemodialysis at our institute was used to attenuate every renal failure case intraoperatively. In our study, hemoglobin less than 10 g/dL was perceived as moderate-severe anemia [11]. Patients’ body mass index (BMI) was obtained with the following equation: weight (kg)/height^2^ (m^2^); accordingly, patients were classified as underweight (BMI < 18.5 kg/m^2^), normal weight (BMI 18.5–24.9 kg/m^2^), overweight (BMI 25.0–29.9 kg/m^2^), obese (BMI 30.0–39.9 kg/m^2^), and morbidity obese (≥ 40 kg/m^2^) [12]. The glucose level measured prior to repair was categorized as follows: ≤140, 141–170, 171–200 and > 200 mg/dl, and patients with glucose >200 mg/dl were diagnosed with severe hyperglycemia [13].

### Endpoint definition

The primary endpoint after aortic replacement was the major adverse events, that is, a composite defined as experiencing one of the following: in-hospital mortality, renal failure, pulmonary infection, reintubation, tracheotomy, arrhythmia, stroke, paraplegia, and gastrointestinal hemorrhage. Other clinical outcomes included time free from mechanical ventilation, and duration of ICU and postoperative in-hospital length of stay.

### Arch replacement technique

All patients in our study underwent TAR with DHCA, which was performed with right axillary and femoral artery cannulation for CPB, antegrade selective cerebral perfusion, and the DHCA technique at 20 °C. This procedure involved implantation of a frozen elephant trunk, total arch replacement with a 4-branched vascular graft (Vascutek Terumo, Tokyo, Japan; 28–30 mm in diameter), a particular sequence for aortic reconstruction (i.e., proximal descending aorta, then left carotid artery, ascending aorta, left subclavian artery, and finally innominate artery), early rewarming and then reperfusion after distal anastomosis to lessen cerebral and coronary ischemia. The duration of selective cerebral perfusion referred to the interval between the initiation of hypothermic circulatory arrest and completion of left carotid anastomosis, which was longer than the duration of DHCA itself. In this period, lower body perfusion was arrested to implant the stented graft (MicroPort Medical Co, Ltd., Shanghai, China; 26–32 mm in diameter) and suture the proximal descending anastomosis.

### Myocardial protection

Blood cardioplegia was used to protect the myocardium in our routine work. Hypothermic techniques combined with ice sprinkled on the surface of the heart were used to achieve the goal of myocardial protection. However, in the surgical process, CPB (S5 roller pump 150, Sorin Group, Munich, Germany) was implemented with tubes not coated; at our institute, coated tube systems were provided to patients in need of extracorporeal membrane oxygenation.

### Intraoperative management

All patients in our center received vasodilator as a routine practice before operation to control systolic pressure under 120 mmHg. In addition, methylprednisolone, an anti-inflammatory agent, was prophylactically administered during the surgical procedure. The use of dexmedetomidine or ulinastatin was determined by the present anesthesiologists and their individual preference towards intraoperative management.

### Statistical analysis

Continuous data were presented as mean and standard deviation (M ± SD) or median and interquartile range (IQR). Categorical data were presented as count and percentage (n, %). For comparisons between cohorts, the t test or the Mann-Whitney U test was used for continuous variables, whereas the chi-square test or Fisher’s exact test was used for categorical variables. Predictive factors for sSIRS were identified with multivariable logistic analysis after collecting baseline characteristics, preoperative biomarker level and perioperative information. A logistic regression model was used to examine the association between sSIRS and major adverse events. All variables with a P-level < 0.2 on univariable analysis were entered into multivariable logistic models. The Kaplan-Meier method was used to estimate the association between severe SIRS and the ICU length of stay. SPSS for Windows release 25.0 (SPSS, Inc., Chicago, IL, USA) was used for all statistical calculations. GraghPad Prism 7.0a was used for the Kaplan-Meier curve.

## Results

### Patient characteristics

**Fig. 1:**
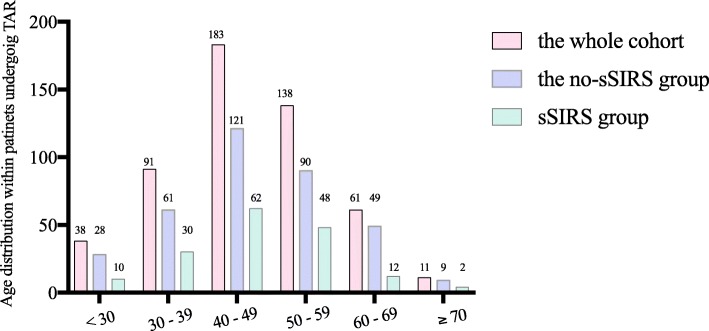
Age distribution of patients undergoing TAR, compared between the whole cohort, the no-sSIRS group and the sSIRS group. The age was 46.7 ± 11.2 years (range, 19–83 years). TAR, total arch replacement; sSIRS, systemic inflammatory response syndrome

A total of 522 patients who underwent TAR with DHCA were included in our cohort. The age was 46.7 ± 11.2 years (range, 19–83 years). The age distribution was depicted in Fig. 1. The weight in this cohort was 74.4 ± 14.1 kg, and the BMI was 25.4 ± 4.9 kg/m^2^. There were 382 (73.1%) males included, and 9.0% had a history of previous cardiac surgery. The number of patients diagnosed with aortic dissection was up to 95.0% (496/522). In all the patient charts, the duration of operation, CPB, aortic cross clamp and DHCA were 378.9 ± 88.4 min, 177.7 ± 50.8 min, 97.3 ± 26.3 min and 21.3 ± 7.0 min, respectively. In addition, following aortic replacement, the time of weaning from mechanical ventilation was 20.0 h (14.0–45.0 h), and the ICU length of stay was 3.0 days (2.0–5.0 days).

### Prevalence of sSIRS

The proportion of patients diagnosed with sSIRS was 31.4% (164/522) within 12–48 h, after they were transferred into the ICU from the operating room.

The comparisons of patients’ baseline characteristics between groups were shown in Table 1. The number of patients aged 60 yr or more was significantly greater in the no-sSIRS group than that in the sSIRS group [14.3% (51/358) vs 6.7% (11/164), *P* = 0.013]. Compared with the no-sSIRS cohort, patients who developed sSIRS after TAR had a lower body weight (*P* = 0.017), higher baseline hemoglobin level (*P* = 0.004) and higher baseline serum creatinine level (*P* = 0.027).

**Table 1:** Comparisons of patient characteristics

Characteristics	Overall (*n* = 522)	sSIRS (*n* = 164)	No-sSIRS (*n* = 358)	*P* value
Baseline				
Male	382 (73.1)	129 (78.6)	253 (70.6)	0.056
Age (years)	46.7 ± 11.2	45.7 ± 10.3	47.2 ± 11.6	0.145
≥ 60	72 (13.8)	14 (8.5)	58 (16.2)	0.018
Weight (kg)	74.4 ± 14.1	76.6 ± 16.4	73.4 ± 12.9	0.017
BMI (kg/m^2^)	25.4 ± 4.9	26.1 ± 4.2	25.4 ± 5.8	0.001
Underweight (< 18.5)	20 (3.8)	6 (3.7)	14 (3.9)	0.126
Normal weight (18.5–24.9)	200 (38.3)	63 (38.4)	167 (46.6)	
Overweight (25.0–29.9)	215 (41.2)	71 (43.3)	144 (40.2)	
Obese (30.0–39.9)	53 (10.2)	21 (12.8)	32 (8.9)	
Morbidly obese (≥ 40)	4 (0.8)	3 (1.8)	1 (0.3)	
Preoperative comorbidities				
Hypertension	365 (69.9)	115 (70.1)	250 (69.8)	0.947
Diabetes mellitus	11 (2.1)	6 (3.6)	5 (1.4)	0.180
Smoking	294 (56.3)	63 (38.4)	131 (36.5)	0.689
Dialysis for renal failure	14 (2.6)	4 (2.4)	10 (2.7)	1.000
Coronary artery disease	23 (4.4)	9 (5.4)	14 (3.9)	0.415
Moderate-severe anemia	20 (3.8)	5 (3.1)	15 (4.2)	0.528
Marfan syndrome	60 (11.4)	20 (12.1)	40 (11.1)	0.734
Aortic dissection	496 (95.1)	154 (93.9)	342 (95.5)	0.073
Aortic aneurysm	26 (4.9)	10 (6.1)	16 (4.5)	0.065
Previous cardiac surgery	47 (9.0)	15 (9.1)	32 (8.9)	0.939
Emergent operation	290 (55.5)	90 (54.8)	200 (55.8)	0.833
Laboratory values				
Hemoglobin (g/dL)	133.3 ± 17.9	136.7 ± 17.6	131.8 ± 17.9	0.004
Serum creatinine (mg/dL)	93.2 ± 36.1	98.4 ± 36.7	90.9 ± 35.5	0.027
Glucose (mmol/l)	6.8 ± 1.6	7.0 ± 1.7	6.8 ± 1.6	0.184
White blood cell count (10^9^/L)	7.2 ± 2.1	7.3 ± 2.2	7.1 ± 2.1	0.235
Echocardiography				
LVEF (%)	59.7 ± 5.5	59.3 ± 6.3	59.9 ± 5.1	0.295
Aortic regurgitation	205 (39.2)	66 (40.2)	139 (38.8)	0.758

Normally distributed continuous data are presented as mean ± SD and categorical data are presented as n (%). *sSIRS* Severe systematic inflammatory response syndrome, *BMI* Body mass index, *LVEF* Left ventricular ejection fraction

Peri-operative information was listed in Table 2. There was no significant difference between groups in the type of concomitant operation, amount of transfusion or the usage of inotropic drugs during surgical repair. The duration of CPB was significantly longer in the sSIRS group [174.0 min (153.3–211.5 min) vs 166.0 min (142.5–197.0 min), *P* = 0.003]. The number of patients administered dexmedetomidine following anesthesia induction was found to be significantly lower in the sSIRS group [85.3% (140/164) vs 93.3% (334/358), *P* = 0.004].

**Table 2:** Peri-operative information between groups

Variables	sSIRS (*n* = 164)	No-sSIRS (*n* = 358)	*P* value
Surgical data			
Concomitant procedures			
Bentall	44 (26.8)	93 (25.9)	0.543
CABG	11 (6.3)	22 (6.1)	0.782
CABG + Bentall	5 (3.0)	9 (2.5)	0.214
Operative Variables			
Operation time (minutes)	376.0 (320.3–448.8)	360.0 (316.0–420.8)	0.213
CPB time (minutes)	174.0 (153.3–211.5)	166.0 (142.5–197.0)	0.003
≤ 180	90 (54.88)	224 (62.57)	0.143
181–240	55 (33.54)	108 (30.17)	
> 240	19 (11.59)	26 (7.26)	
Aortic cross clamp time (minutes)	96.5 (83.3–113.5)	93.0 (79.0–107.3)	0.015
DHCA time (minutes)	21.0 (17.0–25.0)	21.0 (16.0–25.0)	0.288
Lowest temperature (°C)	20.9 ± 3.3	19.5 ± 1.7	0.356
Transfusion			
Red blood cell (u)	2.8 ± 1.2	2.9 ± 1.3	0.857
Fresh frozen plasma (ml)	321 (0–800)	309 (0–1000)	0.638
Platelet (u)	1.7 ± 0.5	1.7 ± 0.6	0.976
Intra-operative inotropic drugs			
Epinephrine	15 (9.2)	31 (8.6)	0.253
Norepinephrine	146 (89.1)	321 (89.6)	0.932
Intra-operative medications			
Ulinastatin	116 (70.7)	273 (76.2)	0.179
Dexmedetomidine	140 (85.3)	334 (93.3)	0.004

Normally distributed continuous data are presented as mean ± SD, whereas non-normal data as median (interquartile range). Categorical data are presented as n (%). *sSIRS* Severe systematic inflammatory systematic syndrome, *CABG* Coronary artery bypass grafting, *CPB* Cardiopulmonary bypass, *DHCA* Deep hypothermic cardiac arrest

### Clinical predictors of sSIRS

The risk factors associated with sSIRS onset with multivariable logistic regression were shown in Table 3. After adjusting for covariates, including sex, age (< 60, ≥ 60 yr), BMI (< 18.5, 18.5–24.9, 25.0–29.9, 30.0–39.9, ≥ 40 kg/m^2^), hyperlipidemia, glucose (≤140, 141–170, 171–200 and > 200 mg/dl), moderate-severe anemia, serum creatinine, smoking, COPD, cerebral infarction, dialysis prior to surgery, hypertension, dissection, coronary disease, cardiac surgery history, aortic regurgitation, left ventricular ejection fraction, CPB duration (< 200, ≥ 200 min), and the usage of ulinastatin or dexmedetomidine, we demonstrated that patients younger than 60 yr had a nearly 3-fold higher likelihood of developing sSIRS (OR = 2.93; 95% CI 2.01–4.28; *P* < 0.001). Higher baseline serum creatinine level prior to surgery contributed to sSIRS development (OR = 1.61; 95% CI 1.18–2.20; *P* = 0.003). The increased risk for sSIRS was 2-fold if patients had concomitant coronary artery disease (OR = 2.00; 95% CI 1.15–3.48; *P* = 0.015). Extended duration of CPB, that is, more than 200 min, was also a risk factor for sSIRS (OR = 1.63; 95% CI 1.23–2.18; *P* = 0.001). However, the intravenous administration of ulinastatin (OR = 0.69; 95% CI 0.51–0.93; *P* = 0.015) or dexmedetomidine (OR = 0.36; 95% CI 0.23–0.56; *P* < 0.001) lowered the risk of sSIRS.

**Table 3:** Risk factors associated with postoperative sSIRS

Variables	Univariable analysis			Multivariable analysis		
	OR	95% CI	P	OR	95% CI	P
Male	1.56	1.17–2.09	0.003			
Age (years)						
<60	1.37	1.06–1.76	0.015	2.93	2.01–4.28	< 0.001
≥ 60	1					
BMI (kg/m^2^)						
Underweight (< 18.5)	0.22	0.05–1.02	0.054			
Normal weight (18.5–24.9)	0.23	0.06–0.83	0.024			
Overweight (25.0–29.9)	0.33	0.09–1.17	0.086			
Obese (30.0–39.9)	0.39	0.10–1.46	0.161			
Morbidly obese (≥ 40)	1		0.011			
Hyperlipidemia	1.18	0.90–1.56	0.231			
Glucose (mmol/l)						
(≤ 140)	1.61	0.53–4.87	0.397			
(141–170)	2.21	0.72–6.83	0.168			
(171–200)	1.58	0.45–5.50	0.473			
(> 200)	1		0.161			
Moderate-severe anemia	1.51	0.80–2.84	0.200			
Serum creatinine	1.66	1.23–2.24	0.001	1.61	1.18–2.20	0.003
Smoking	1.02	0.79–1.33	0.868			
COPD	1.62	0.54–4.93	0.392			
Cerebral infarction	0.77	0.47–1.28	0.320			
Dialysis prior to surgery	1.24	0.55–2.79	0.609			
Hypertension	0.98	0.73–1.32	0.915			
Dissection	1.15	0.64–2.04	0.647			
Coronary disease	0.69	0.42–1.14	0.151	2.00	1.15–3.48	0.015
Cardiac surgery history	0.78	0.67–1.70	0.784			
Aortic regurgitation	0.99	0.76–1.28	0.920			
LVEF	0.98	0.96–1.01	0.118			
CPB (minutes)						
< 200	1					
≥ 200	1.59	1.21–2.09	0.001	1.63	1.23–2.18	0.001
Ulinastatin	0.63	0.48–0.83	0.001	0.69	0.51–0.93	0.015
Dexmedetomidine	0.36	0.24–0.54	<0.001	0.36	0.23–0.56	< 0.001

*sSIRS* Severe systematic inflammatory systematic syndrome, *OR* Odds ration, *CI* Confidential interval, *BMI* Body mass index, *COPD* Chronic obstructive pulmonary disease, *LVEF* Left ventricular ejection fraction, *CPB* Cardiopulmonary disease

### Clinical outcomes associated with sSIRS

In-hospital outcomes were listed in Table 4.

**Table 4:** In-hospital outcomes after TAA with DHCA

Variables	sSIRS (*n* = 164)	No-sSIRS (*n =* 358)	*P* value
Major adverse events	93 (56.7)	96 (26.8)	< 0.001
In-hospital mortality	8 (4.8)	4 (1.1)	0.019
Renal failure	33 (20.1)	28 (7.8)	< 0.001
Pulmonary infection	67 (40.8)	71 (19.8)	< 0.001
Re-intubation	16 (9.7)	10 (2.7)	0.001
Tracheotomy	11 (6.7)	2 (0.5)	< 0.001
Arrhythmia	32 (19.5)	26 (7.2)	< 0.001
Stroke	6 (3.6)	5 (1.4)	0.180
Paraplegia	9 (5.4)	9 (2.5)	0.084
Gastrointestinal hemorrhage	10 (6.1)	7 (1.9)	0.013
Re-thoracotomy for bleeding	8 (4.8)	11 (3.0)	0.307
Mechanical ventilation time (hours)	82.6 (23.2, 235.4)	37.4 (13.0, 102.3)	< 0.001
Intensive care unit length of stay (days)	7.0 (3.0, 10.0)	3.0 (1.0, 7.0)	< 0.001
Length of hospital stay (days)	14 (7.0, 24.0)	12.0 (6.0, 19.0)	< 0.001

*TAA* Total arch replacement, *DHCA* Deep hypothermic cardiac arrest, *sSIRS* Severe systematic inflammatory systematic response

### Major adverse events

The proportion of major adverse events was significantly greater in patients suffering sSIRS compared with the no-sSIRS cohort [56.7% (93/164) vs 26.8% (96/358), *P* < 0.001]. There were 8 deaths (4.8%) in the sSIRS group and 4 (1.1%) in patients without sSIRS, which was a significant difference (*P* = 0.019). With logistic regression adjusting for related-covariates, sSIRS still had a 4.5-fold increased risk of occurrence of any major adverse event following TAR (OR, 4.52; 95% CI, 3.40–6.01; *P* < 0.001). Age greater than 60 yr (OR, 1.81; 95% CI, 1.32–2.48; *P* < 0.001), severe hyperglycemia (OR, 3.48; 95% CI, 1.35–8.97; *P* = 0.01), dialysis to treat renal failure prior to surgery (OR, 2.21; 95% CI, 1.05–4.68; *P* = 0.038), emergent status (OR, 1.52; 95% CI, 1.17–1.98; *P* = 0.002) and duration of CPB longer than 200 min (OR, 2.83; 95% CI, 2.12–3.77; *P* < 0.001) were also independently associated with major adverse events, as seen in Table 5.

**Table 5:** Multivariable logistic regression for major adverse events following arch replacement

Variables	Univariable analysis			Multivariable analysis		
	OR	95% CI	*P*	OR	95% CI	*P*
Male	0.94	0.73–1.22	0.650			
Age (years)						
< 60	1					
≥ 60	1.31	0.99–1.75	0.058	1.81	1.32–2.48	< 0.001
BMI (kg/m^2^)						
Underweight (< 18.5)	0.50	0.11–2.30	0.374			
Normal weight (18.5–24.9)	0.96	0.27–3.45	0.952			
Overweight (25.0–29.9)	1.09	0.30–3.93	0.892			
Obese (30.0–39.9)	0.94	0.25–3.53	0.930			
Morbidly obese (≥ 40)	1		0.447			
Severe hyperglycemia	2.29	0.93–5.65	0.092	3.48	1.35–8.97	0.010
Dissection	0.55	0.30–1.01	0.053			
Hypertension history	1.48	1.11–1.97	0.007			
Preoperative renal failure	1.86	0.91–3.81	0.090	2.21	1.05–4.68	0.038
Coronary artery disease	2.05	1.25–3.34	0.004			
Emergent operation	1.31	1.03–1.66	0.029	1.52	1.17–1.98	0.002
CPB (minutes)						
< 200	1					
≥ 200	2.81	2.15–3.67	< 0.001	2.83	2.12–3.77	< 0.001
sSIRS	4.01	3.11–5.30	< 0.001	4.52	3.40–6.01	< 0.001

*OR* Odds ratio, *CI* Confidence interval, *BMI* Body mass index, *CPB* Cardiopulmonary bypass, *sSIRS* Severe systematic inflammatory systematic response.

### Other in-hospital outcomes

**Fig. 2:**
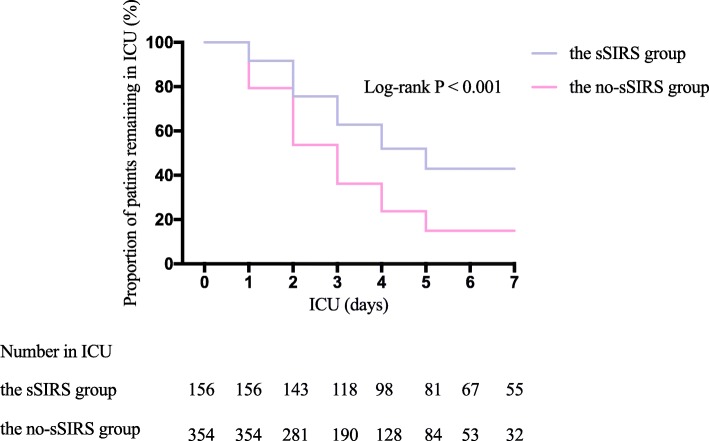
The comparison of ICU length of stay with the Kaplan-Meier curve. The proportion of patients treated in the ICU following TAR was significantly greater in the sSIRS group compared with patients without sSIRS (log-rank *P* < 0.001) after excluding 12 in-hospital deaths. sSIRS, severe systematic inflammatory response syndrome; ICU, intensive care unit; TAR, total arch replacement. ICU, intensive care unit; TAR, total arch replacement; sSIRS, systemic inflammatory response syndrome

Patients with sSIRS were characterized by longer mechanical ventilation duration (*P* < 0.001), ICU length of stay (*P* < 0.001) and postoperative hospital stay (*P* < 0.001). The percentage of patients experiencing prolonged ICU duration in the sSIRS group (> 7 d) was significantly greater than that in the no-sSIRS group [36.0% (59/164) vs 9.8% (35/358), *P* < 0.001]. The Kaplan-Meier curve indicating the time to the discharge from the ICU was depicted in Fig. 2. The median time to discharge from the ICU was 5.0 days (95% CI 4.2–5.8 days) in the sSIRS group and 3.0 days (95% CI 2.8–3.2 days) in the no-sSIRS group, log-rank *P* < 0.001.

## Discussion

To the best of our knowledge, this is the first study to investigate the prevalence of severe inflammation following TAR with DHCA. The incidence of sSIRS was 31.4% following TAR regardless of routine methylprednisolone prophylaxis, which obviously contributed to the postoperative development of major adverse events.

The inflammatory response after cardiac surgery has been widely recognized [4, 14, 15]; however, few studies have focused on its occurrence following repair of aortic pathology, especially its severity. SIRS, defined when the patient met two or more criteria, is seen in 96.2% of patients after cardiac surgery, without any discriminatory value for predicting clinical outcomes [6]. A previous study carried out among patients undergoing elective abdominal aneurysm repair demonstrated that SIRS development was as high as 89% [16]. Recently, Lindman and colleagues [2] introduced the application of severe SIRS, which developed in 11% of patients undergoing surgical aortic valve replacement but 6% of patients treated with transcatheter aortic valve replacement; sSIRS itself was also strongly associated with a greater risk of six-month mortality. It is the conception of severe SIRS rather than conventional SIRS criteria that is more appropriate to depict the relationship between the inflammatory response and its clinical outcomes.

The patients undergoing arch repair for aortic pathology had an average age of 40 yr at our institute, younger than those of other investigations, where patients aged 53.9 to 70.1 yr [17–21]. The incidence of emergent status was as greater, at 55.8%, than in previous settings. At two institutes in Japan, the emergency rates were 26.1% [17] and 25.1% [18], respectively, according to recent publications in the field of TAR using a 4-branched graft. The definition of an emergent procedure at our institution was within 24 h prior to surgery, while the precise time was not given in those two single-center experiences.

In this study, we demonstrated the clear association of age with sSIRS development. Our logistic regression showed that advancing age was correlated with a lower risk of sSIRS in patients following TAR with DHCA. Elderly individuals presented delayed initiative and even poor maintenance with regard to the immune system after encountering inflammatory stressors [22]. It is a fact that age-related immunosenescence, consisting of dysregulation of immune cells (such as incompetency to generate pro-inflammatory cytokines and compromising capacity of phagocytizing) and reduced level of C-reactive protein upon interleukin-6 stimulation is a common scenario in elderly patients [23–25]. This kind of nonintensive inflammation reaction uniquely linked to advancing age has strong evidence in clinical work. There was striking evidence that the risk of SIRS at any time within 24 h after cardiac surgery is uniquely attenuated in patients aged 72 yr or older [4]. It is hypothesized that the phenotypic and genomic variation and patients’ susceptibility had good perioperative predictability of the individual inclination to develop inflammatory syndromes. Systematic inflammatory responses were also not intensive and diminished in patients aged over 80 yr who had community-acquired pneumonia and reduced levels of C-reactive protein and cytokines after admission [26]. A precise scheme for anti-inflammation should be put into practice in our clinical settings, although the patients’ age varies greatly. The therapeutic approach, such as intraoperative dexamethasone, gave no benefit to patients aged over 80 yr undergoing cardiac repair [27].

This study showed that either dexmedetomidine or ulinastatin could diminish the likelihood of postoperative development of sSIRS. Dexmedetomidine, a highly selective α_2_-adrenergic agonist and universal option for sedation, has been revealed to be effective in reducing cytokine release associated with the nuclear factor kappa B activation inhibition mechanism in cardiac surgery with CPB [28, 29]. However, whether dexmedetomidine is of prophylactic benefit in a population with sSIRS has been obscure, and more clinical trials are imperative in the future. Ulinastatin, extracted from humane urine, acts as a unique anti-inflammatory agent with a mechanism that includes the inhibition of neutrophil elastase and of various other proteases. Clinical trials have provided robust evidence that its administration in cardiac surgery with CPB could attenuate postoperative typical inflammatory biomarker release, such as interleukin, tumor necrosis factor-α, and other cytokines [30–32]. Consequently, the organ-protective property of ulinastatin has been reported, primarily in correlation with attenuating acute kidney injury, pulmonary compromise and hemodynamic instability [30, 33].

Patients diagnosed with sSIRS were found to have a greater likelihood of suffering any adverse complications after the TAR procedure. In-hospital outcomes were not promising in the sSIRS cohort: they had extended duration of weaning from mechanical ventilation, as well as prolonged duration of ICU length of stay and postoperative hospital stay. In a population with transcatheter aortic valve implantation, sSIRS has also raised the risk of certain adverse events: mortality, stroke, infection, bleeding, myocardial infarction and acute kidney injury [3]. It is implied that sSIRS also has predictive ability for the length of stay after admission to the ICU [9].

Undergoing an emergent procedure could have enhanced the chance of experiencing major adverse events in patients following TAR in our investigation. One of the potential reasons would be that the population requiring emergency treatment was indeed in an exacerbated status prior to surgery and had greater risks to develop sequentially worse outcomes after the implementation of total arch repair. However, another possibility, which cannot be ruled out, is that preoperative temporary treatments for patients were insufficient within the limited duration under study, so that they did not reach an optimized status and then had a higher incidence of major adverse events after discharge from the operating room.

No further analysis of neurological defects following TAR, such as stroke or paraplegia until hospital discharge, was carried out in this cohort study owning to their lower incidence (2.1, 3.4%, respectively). Previous investigations provided robust evidence that stroke primarily occurred in patients with concomitant coronary artery bypass grafting, cerebrovascular defect history, or new-set atrial fibrillation [34, 35]. Surgical techniques, including hypothermic circulatory arrest times, selective antegrade cerebral temperature [36, 37], unilateral or bilateral cerebral perfusion [38] and treatment for distal aortic arch aneurysm [19], also significantly contribute to stroke development. There is a classic view in clinics that coronary artery disease is an alternative to atherosclerosis, feasibly extending to the bloodstream and maximizing the risk of thrombosis of the neurologic system. Paraplegia, a serious complication of spinal injury, can be primarily predicted by stented elephant trunk implantation. Extended stent graft implanted into the descending aorta could harm the intercostal arteries, and then collateral blood supplying to the spinal cord extremely deteriorates [20]. Therefore, cerebral spinal fluid drainage, and reduced hypothermic circulatory arrest to 25 °C, and stent-graft lengths less than 10 cm are practical techniques to prevent spinal cord injury [18, 20].

This study has several limitations. First, heterogeneity existed owning to its retrospective and single-centered nature. Second, the identification of SIRS and even its severity following TAR may not be accurate because it was only judged by patients’ vital signs instead of strong evidence from serial biomarkers measurements, including serum C-reactive protein and interleukins. The number of leukocytes and the ratio of lymphocytes/macrophages cannot be obtained routinely, major contributors to the imperfection of this work. Third, it was proposed that meeting three criteria of SIRS within 24 h after cardiac surgery or at least two criteria for 6 h would be more valid in predicting clinical outcomes [6]. However, the time for severe SIRS in our research was extended until 48 h postoperatively. The validity of sSIRS for organ dysfunction, ICU length of stay and hospital stay should be explored further. Forth, evidence of preoperative malperfusion cannot be obtained, which resulted in bias. Finally, all participants with aortic dissection undergoing repair had a history of acute status. Further investigations should be aimed at elucidating the relationship between sSIRS onset and acute, subacute or chronic dissection pathology.

## Conclusion

sSIRS is as common as 31.4% in patients following total arch replacement with the DHCA technique. There was an inverse association between age over 60 yr and sSIRS onset. Higher baseline serum creatinine, concurrent coronary disease and prolonged CPB time aggressively contribute to a greater possibility of postoperative sSIRS, while the preferred administration of ulinastatin and dexmedetomidine can attenuate it. sSIRS has significant predictive value for postoperative major clinical adverse events, which can also be increased by severe hyperglycemia, concomitant kidney failure prior to repair, emergent procedure and extended duration of CPB.

## Data Availability

The datasets used and/or analyzed during the current study are available from the corresponding author on reasonable request.

## Abbreviations

BMI: Body mass index
CI: Confidence intervals
CPB: Cardiopulmonary bypass
DHCA: Deep hypothermic circulatory arrest
ICU: Intensive care unit
IQR: Interquartile range
IRB: Institutional Review of Board
KDIGO: Kidney Disease: Improving Global Outcomes
OR: Odds ration
PaCO_2_: Partial pressure of arterial carbon dioxide
SD: Standard deviation
sSIRS: Severe systemic inflammatory response syndrome
TAR: Total aortic arch replacement
